# Mitochondrial DNA population variation is not associated with Alzheimer’s in the Japanese population: A consistent finding across global populations

**DOI:** 10.1371/journal.pone.0276169

**Published:** 2022-10-20

**Authors:** Johanna Wong, Jannetta S. Steyn, Ilse S. Pienaar, Joanna L. Elson

**Affiliations:** 1 The Bioscience Institute, Newcastle University, Newcastle upon Tyne, United Kingdom; 2 Research Software Engineering, Newcastle Helix, Newcastle upon Tyne, United Kingdom; 3 Institute of Clinical Sciences, University of Birmingham, Edgbaston, United Kingdom; 4 The Centre for Human Metabolomics, North-West University, Potchefstroom, South Africa; Institut d’Investigacions Biomediques de Barcelona, SPAIN

## Abstract

Several mitochondrial DNA (mtDNA) haplogroup association studies have suggested that common mtDNA variants are associated with multifactorial diseases, including Alzheimer’s disease (AD). However, such studies have also produced conflicting results. A new mtDNA association model, the ‘variant load model’ (VLM), has been applied to multiple disease phenotypes. Application of the VLM in a 2017 study failed to find different variant loads in AD patients compared to controls, in two cohorts of European origin. The study also suggested a lower variant load in healthy elderly individuals, but could offer no replicate cohort to support this observation. Here, the VLM is applied to Japanese mtDNA sequences; in doing so, we explored the role of mtDNA variation in AD and ageing in a different global population. Consistent with the previous findings using the VLM in two populations of European origin, we found no evidence for an association between rarer, non-haplogroup associated variation and the development of AD. However, the result in the context of ageing that suggested those with fewer mildly deleterious mutations might undergo healthier ageing, was not replicated. In contrast to our previous study, our present results suggest that those living to advanced old age may harbour more mildly deleterious mtDNA variations. Importantly our analysis showed this finding is not primarily being driven by many rare population variants dispersed across the mtDNA, but by a few more frequent variants with high MutPred scores. It is suggested the variants in question do not exert a mildly deleterious effect in their most frequent haplogroup context.

## Introduction

Mitochondrial DNA (mtDNA) is 16.6 kilobases in length, encoding for 37 genes, including 13 polypeptides which form core subunits of 4 out of the 5 oxidative phosphorylation (OXPHOS) protein complexes. MtDNA population variants have been implicated in multifactorial diseases, including hypertension, diabetes, atherosclerosis and Alzheimer’s disease (AD) [1–3]. Hypotheses linking mtDNA variation to the underlying neuropathology of AD include excessive oxidative stress due to mildly deleterious variants [4]. Reduced activity in Complex IV has also been highlighted by a number of investigators [5]. MtDNA is maternally inherited, meaning that the evolution of mtDNA is defined by the emergence of distinct global lineages called haplogroups. Different common mitochondrial haplogroups have been associated with increased (and decreased) risk of AD in various studies; some studies have failed to find any associations at all, including a recent meta-analysis [6]. The most likely reason for these conflicts in the literature is population stratification that occurs with common mtDNA variants [7].

In response to the conflicting data generated by the haplogroup association model, the variant load model (VLM) has recently been introduced [1, 8–10]. The VLM investigates the combined effects of population variants predicted to be mildly deleterious, most of which are rare. Rare variants do not show the same geographical stratification as common variants. We recently applied the VLM to investigate AD patients compared to aged, but non-neurodegenerative disease patients, using sequences from two independent cohorts, the MitoKor (MK-US) and Medical Research Council (MRC-UK) cohorts [1, 11]. The study found no association between mtDNA variant load and the onset of AD. Surprisingly, the variant load was lower in the US controls, indicating a potential association between low VL and healthy ageing. In order to validate these results in a different population, the current analysis explored the role of rare mtDNA variants in Japanese cohorts, consisting of both AD patients and the aged, but otherwise healthy individuals and a younger cohort.

## Methods

### Sequence data

Sequence data were extracted from the mitochondrial single nucleotide polymorphism (mtSNP) database (http://mtsnp.tmig.or.jp/mtsnp), which is compiled by the Gifu International Institute of Biotechnology–The Japanese Science and Technology Agency (GiiB-JST) [9]. The sequences were all taken from Japanese individuals described as: 96 centenarians, 96 Alzheimer’s disease patients (mean age 76.5 (+/- 9.7) years) and 96 healthy non-obese young males (mean age 20 (+/- 3) years. The centenarians, by definition, were 100 years old or more. The data used in this analysis was not accompanied by information regarding the heteroplasmy levels. The sequencing was conducted using Sanger methodology all the variants in this study can be considered operationally homoplasmic.

### Data management and statistical analysis

We estimated the pathogenicity or mildly deleterious effect of mtDNA variants by using the MutPred web application (http://mutpred1.mutdb.org). Protein encoding variants with a score of >0.5 were included in the analysis as they are suggested to have a mildly deleterious effect on protein function [12]. We analysed the transfer RNA (tRNA) genes in the 3 cohorts using MitoTIP, an in silico tool for tRNA pathogenicity prediction, which is embedded into MITOMASTER, a human mtDNA sequence analysis tool that is based within the MITOMAP human mitochondrial genome database (http://www.mitomap.org) [13, 14]. For statistical analysis we performed a one-way ANOVA and Tukey *post-hoc* test, using SPSS Statistics software (version 24.0 (released in 2016); IBM Corporation, Armonk, USA).

## Results

### Haplogroup distribution of study sequences

Our dataset comprised of a haplogroup mixture that is generally in line with the expected haplogroup frequency in Asia, comprising sequences from haplogroups A, B, C, D, F, G, M, N, Y and Z, with predominance of haplogroups D4, M7 and B4 and N9 [9, 15].

### Variant load comparison

The calculated mean “all scoring variants”, “MutPred >0.5 variants”, and “MitoTIP tRNA variants” variant loads for each group are shown in Table 1. MutPred >0.5 Variant Load Comparison.

**Table 1:** Mean variant loads (with standard deviations) for Japanese centenarian, AD patient and healthy young cohorts, calculated using: (a) all MutPred scoring variants, (b)variants scoring >0.5 on MutPred and (c) MitoTIP tRNA variant scores.

	Study Cohort	Mean Variant Load	Maximum single sequence variant load	Minimum single sequence variant load	Number of sequences with no relevant variants
**All scoring variants, all genes (a)**	Centenarians	2.6213 (+/-0.77)	4.735	0.928	0
	AD	2.4080 (+/-0.69)	4.232	0.947	0
	Non-obese young	2.4349 (+/-0.75)	4.342	0.947	0
**MutPred >0.5, all genes (b)**	Centenarians	0.7537 (+/-0.64)	2.827	0	24
	AD	0.5670 (+/-0.51)	2.425	0	28
	Non-obese young	0.5768 (+/-0.51)	2.425	0	27
**tRNA MitoTIP scores (c)**	Centenarians	5.0093 (+/-11.08)	78.9	0	56
	AD	6.5365 (+/- 13.71)	81.3	0	51
	Non-obese young	7.4083 (+/- 12.71)	68.7	0	48

MutPred predicts the likely deleteriousness of amino acid substitutions base on their impact on protein structure and function, as well as evolutionary conservation, scoring variants from 0 to 1. The tRNA variant load figures are 2 orders of magnitude larger than the MutPred protein-coding variant loads as MitoTIP generates scores in percentages, based on conservation, database frequencies or variants and the nature of the nucleotide change.

**Fig 1:**
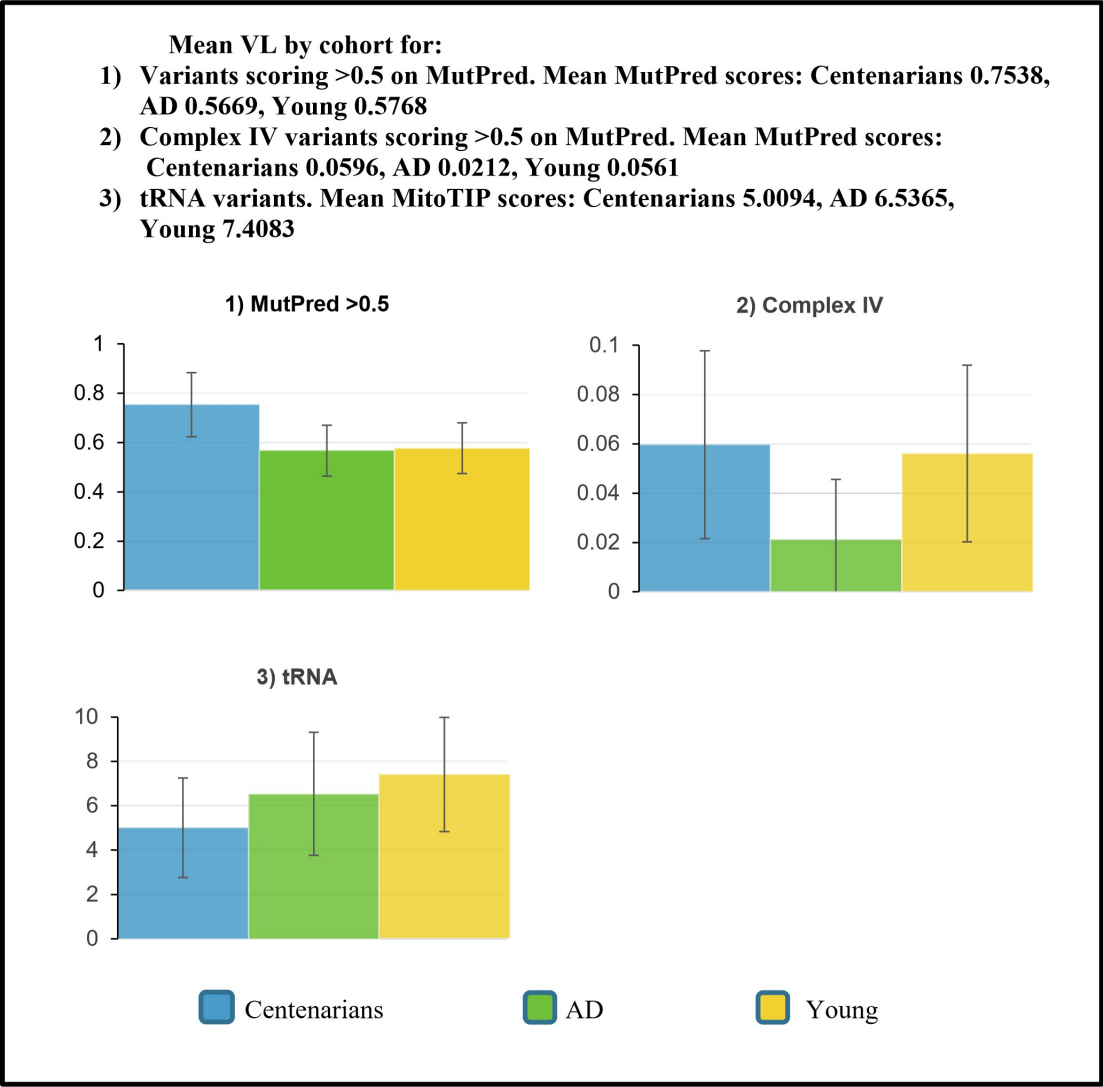
Means and 95% confidence intervals of variant loads calculated using MutPred. All genes variants scoring >0.5 on MutPred (top left), variants scoring >0.5 on MutPred affecting Complex IV (top right) and MitoTIP tRNA variants (bottom left).

When considering all genes at a MutPred variant load setting of >0.5, a statistically significant difference was found between the three groups (Fig 1) (*F* (2.12, 88.03) = 3.43, *p* = 0.034). A Tukey *post-hoc* test revealed a statistically significantly higher all genes MutPred >0.5 variant load in centenarians (mean variant load = 0.754, SD +/- 0.641, *p* = 0.054) compared with the AD group (mean variant load = 0.567, SD +/- 0.508); with a difference in the healthy young group (mean variant load = 0.577, SD +/- 0.508, *p* = 0.07). This finding, of higher variant loads in centenarians (albeit of marginal significance) opposes our earlier findings [11]. Refining the analysis to utilise just the MutPred scores of variants in genes affecting Complex IV scoring >0.5, again found no statistically significant difference between the groups (F (0.09, 7.71) = 1.60, p = 0.203). Supplemental Tables (a-c) provide all raw variant load values by sequence.

### Transfer RNA variant load comparison

The centenarian mean tRNA variant load value was the lowest of the 3 cohorts (Fig 1). However, no statistically significant difference was found between the groups, following application of a one-way ANOVA (*F* (2, 285) = 0.899, *p* = 0.41). Within this sub-analysis, over 50% of the analysed sequences produced a null score.

### Haplogroup context of high scoring variants

Interrogation of the centenarian sequences revealed 9 high-scoring variants (>0.5), which occurred more than twice in this cohort. A number of these variants (3391A, 5460A, 8414T, 8794T, 12811C and 13651G) appeared to be present in higher frequencies in the centenarian cohort than in the other 2 groups. Table 2 shows the variants, along with their more common haplogroup associations using MITOMAP data from 2019. Importantly, none of these high-scoring variants appeared to be present out of the normal haplogroup context. In the majority of cases, the variants were present in >98% of the analysed sequences of the corresponding haplogroup branch, as seen in GenBank. The remaining variants were not particularly uncommon in their haplogroup contexts, being present in >10% of the relevant GenBank sequences.

**Table 2:** Haplogroup context and common GenBank 2019 Haplogroup associations of variants scoring >0.5 in MutPred found in the study population.

Variant	Haplogroups in which present in study	Centenarians by top level haplogroup	Total	AD by top level haplogroup	Total	Young by top level haplogroup	Total	MutPred score	GenBank clades	GenBank sequence count	GenBank haplogroup branch frequency (%)
**8414T**	D4a D4b D4c D4e D4f D4g D4h D4j D4i D4m D4n	8 19 5 3 0 2 0 0 1 1 0	39	5 13 3 3 1 2 2 0 1 0 1	31	4 12 3 5 1 5 0 1 1 0 1	33	0.513	D1 D1f D2a D4 D4a **D4b** D4c D4e D4g D4h D4j D4o	204 50 63 56 146 **232** 81 149 77 113 238 53	99.51 100.00 100.00 96.55 100.00 **99.57** 100.00 100.00 100.00 99.12 99.58 100.00
**5460A**	A5a D4j G4 M7b N9a	1 1 0 7 0	9	0 0 0 4 1	5	0 0 1 1 0	2	0.505	H1e J1b K1a L0a L0d **M7b** W1 W3a	246 227 54 672 79 **594** 69 91	99.6 66.18 5.26 99.85 12.04 **98.84** 100.00 98.91
**8794T**	A5a A5c A7 A25	7 0 1 0	8	3 1 0 1	5	2 2 0 0	4	0.608	A A15c A2 A2a **A5a**	58 52 331 226 **65**	93.31 100.00 99.7 100.00 **100.00**
**8584A**	B5a B5b C1a M8a Z4a	3 2 1 0 1	7	1 4 0 1 0	6	0 1 0 2 0	3	0.553	B5a **B5b** C C1b C1c C1d C4a C4b C7a M8a Z1a	380 **145** 436 119 57 110 351 165 141 66 78	100.00 **100.00** 99.54 99.17 99.61 100.00 97.77 99.4 100.00 97.06 98.73
**12811C**	M7b	7	7	4	4	1	1	0.587	H3h **M7b**	65 **508**	100.00 **84.53**
**13651G**	D4a	5	5	0	0	1	1	0.626	**D4a** L3e	**18** 96	**12.33** 10.95
**3391A**	D4c	4	4	1	1	2	2	0.862	**D4c**	**17**	**20.99**
**15662G**	B5b	4	4	4	4	1	1	0.753	**B5b**	**145**	**100.00**
**12880C**	A5a	3	3	3	3	1	1	0.635	**A5a**	**30**	**46.15**
**Total variants occurring 2+ times in centenarian cohort, by cohort**		**Centanarians**	**86**	**AD**	**59**	**Young**	**48**				

Frequency in each of the study cohorts of variants occurring more than twice in the centenarian cohort with their sequence haplogroup contexts.

Variants listed in order of frequency in the centenarian cohort.

Top level haplogroups as listed on GiiB-JST mtSNP database(http://mtsnp.tmig.or.jp/mtsnp). GenBank clades listed are those containing the variants with a GenBank sequence count of 50 or greater. If there are none with a count more than 50, then clades seen in the majority of the study population and the most numerous relevant GenBank clade, if different, are listed. Bold type denotes the haplogroups of the majority of sequences carrying each variant in the study cohorts.

## Discussion

Several studies have implicated mtDNA variation in the onset and progression of AD. Here we explored the role of mtDNA variation in the onset of AD using a new association strategy, the VLM, applied to an East-Asian population dataset [15]. Our results do not provide any evidence for an association between levels of mildly deleterious mtDNA and the onset of AD. This finding also supports our prior observations, made in UK and US-based populations [1, 11]. In contrast to our previous study, our present results suggest that those living to advanced old age may harbour more mildly deleterious mtDNA variations. While, intuitively, it seems logical that fewer mildly deleterious mutations might associate with healthier ageing, some experimental evidence suggest that a limited degree of mitochondrial functional decline can lead to a lengthened lifespan in a range of organisms, including *C*. *Elegans*, *Drosophila* and mice [16, 17].

Our current study cohort and that used in our previous study, have some notable differences, as shown in S1 Table. Firstly, the elderly group differed in age, with the average age of the aged controls in the study by Pienaar and others (2017) that were 77.2 years (± 9.6, Standard Deviation (SD)) for the UK cohort and 83.4 years (± 5, SD) in the US cohort. In contrast, the Japanese centenarians were, by definition, more than a decade older. As little information about the sequenced individuals is available from either the database website or the two original study papers, little is known about the health status of the centenarians or the criteria used for AD diagnosis. This suggests that a better approach might be to apply such a model in more comprehensively described ageing cohort. There are a number of cohorts including those of octogenarians from Newcastle (UK) and Leiden (Netherlands) with detailed phenotypic data gathered at baseline then 3 and 5 years. As being able to correlate with the specific aspects of the health, status of populations with mtVLM would provide a much more satisfactory link of genotypes to phenotype.

The definitions used to classify the healthy non-obese young men are also unclear, although their mean body mass index (BMI) was 20.2 (+/- 2.3).

Although the aim in using the VLM was to ameliorate the effects of population stratification, a residual stratification effect may influence the results of VLM studies, due to a very small number of common haplogroup-defining variants remaining, even when only using variants with MutPred scores >0.5 for the analysis [3]. In the present work, we also demonstrated the presence of haplogroup-defining variants in this Asian population with high MutPred scores, suggesting that they may be mildly deleterious. In the centenarian cohort, who had the highest mean variant load scores, nine individuals had frequently occurring (more than twice) variants, with high MutPred scores. Of these variants, five are present in over 98% of the GenBank haplogroup sequences corresponding to the most common haplogroups of the study sequences in which they were found. Two of these (8414T and 8794T) are listed as markers for their respective haplogroups on MITOMAP, while another (8584A) is listed as a marker of the major branch of the mtDNA phylogenetic tree. The other cohorts also had frequently occurring high-scoring variants; ten in the AD group and five in the healthy young group.

Overall, our analysis suggests that our finding of a marginally higher variant load in centenarians is not primarily being driven by many rare population variants, but by a few common variants that have high MutPred scores. We predict that these variants do not exert a deleterious effect due to the haplogroup context in which they are found. In support of this a number of studies have been completed that have found mutations shown to cause inherited mitochondrial disease in humans to be present in other species in the absence of disease [18–20]. These investigations made suggestions as to what elements of the haplogroup context of the human mutations that allowed them to exist as benign variants in these species. Here we make the speculation that if the deleterious effect of very rare population variants that have been casually linked to inherited disease can be compensated by haplogroup background, so can the effect of mildly deleterious population variants in the context of common complex disease. Additional support of this notion comes from work to suggest common population variants seen in a different haplogroup context (in humans) might be associated with the onset of common disease [21]. We explored this in the current work with none of the high-scoring variants in centenarians being seen out of their usual haplogroup context. Population variants that are haplogroup associated when seen in a different haplogroup context have been called private variants in the past and are an area of interest in the context of mtDNA population variation and common disease [1]. The use of this terminology again might be of help in the development of new models. Hence, a “shadow” of the haplogroup stratification effect may endure in VLM-based studies, potentially contributing to inconsistent results. Taken together this suggests that refinement to the VLM might be required if it is to achieve its aim. But this will may not be as simple as the elimination of frequently occurring variants with high MutPred scores. As recent paper, patients with atherosclerosis were found to have a higher likelihood of having a common variant scoring >0.5 in MutPred, alongside a rarer variant with a similarly high score. This suggests a two-hit hypothesis and [8].

In summary, a consistent null picture in the context of the role of mtDNA variation in AD is emerging [11, 22], after application of a number of methods for investigating this notion. Further exploration of mtDNA variant load association in the context of ageing would benefit from larger, dedicated cohorts, consisting of individuals with detailed clinical and demographic information this approach has been used in other phenotypes [2, 10] such cohorts exist in the ageing context but as yet mtDNA sequence data is unavailable [23, 24].

## Supporting information

S1 Table Comparative features and variant loads of aged cohorts in this study and in Pienaar et al. **(2017).** Illustrates the mean “MutPred >0.5 variants” variant load and the mean “all scoring variants” variant load for the aged cohorts in both studies. Although the older MitoKor group appeared to have lower mean variant loads than the MRC group, analysis of the even older GiiB-JST centenarians did not produce similarly lowered mean variant loads.
(DOCX)
