# Supplementary material for: Mitochondrial DNA population variation is not associated with Alzheimer’s in the Japanese population: A consistent finding across global populations
Source: PLoS One. 2022 Oct 20;17(10):e0276169. doi: 10.1371/journal.pone.0276169 (PMC9584534; doi:10.1371/journal.pone.0276169)
Supplement: S1 Table — (2017). Illustrates the mean “MutPred >0.5 variants” variant load and the mean “all scoring variants” variant load for the aged cohorts in both studies. Although the older MitoKor group appeared to have lower mean variant loads than the MRC group, analysis of the even older GiiB-JST centenarians did not produce similarly lowered mean variant loads. (DOCX) [file pone.0276169.s001.docx]

**Supplemental Table 1.**

|  | **Pienaar et al. (2017)** | | **Current Study** |
| --- | --- | --- | --- |
| **Cohort designation** | MitoKor controls | MRC controls | GiiB-JST  centenarians |
| **Number of individuals** | 64 | 64 | 96 |
| **Location** | USA | UK | Japan |
| **Age (Years)** | 83.4 | 77.2 | 100+ |
| **Haplogroups** | European H,V,U,K,T,J,W, I, X | European H,V,U,K,T,J,W, I, X | Asian A,B,C,D,F,G,M,N,Y,Z |
| **Mean Variant Load** | 1.456  (SD +/- 0.78) | 2.273  (SD +/- 0.92) | 2.6213  (SD +/- 0.77) |
| **Mean MutPred >0.5 Variant Load** | 0.3798  (SD +/- 0.47) | 0.86  (SD +/- 0.71) | 0.7537  (SD +/- 0.64) |
